# Retracted: Securitization Concept and Its Application to Environmental Problems in the Kurdistan Region: Prospects and Obstacles

**DOI:** 10.1155/2023/9867104

**Published:** 2023-03-09

**Authors:** Journal of Environmental and Public Health

*Journal of Environmental and Public Health* has retracted the article titled “Securitization Concept and Its Application to Environmental Problems in the Kurdistan Region: Prospects and Obstacles” [1] due to concerns that the peer review process has been compromised.

Following an investigation conducted by the Hindawi Research Integrity team [2], significant concerns were identified with the peer reviewers assigned to this article; the investigation has concluded that the peer review process was compromised. We therefore can no longer trust the peer review process, and the article is being retracted with the agreement of the Chief Editor.

Nusret Sinan Evcan agrees to the retraction; Salam Abdulqadir Abdulrahman was unresponsive.
